# Preliminary Evaluation of 3D Printed Chitosan/Pectin Constructs for Biomedical Applications

**DOI:** 10.3390/md19010036

**Published:** 2021-01-15

**Authors:** Georgia Michailidou, Zoe Terzopoulou, Argyroula Kehagia, Anna Michopoulou, Dimitrios N. Bikiaris

**Affiliations:** 1 Laboratory of Polymer Chemistry and Technology, Department of Chemistry, Aristotle University of Thessaloniki, 555 35 Thessaloniki, Greece; gmichailid@chem.auth.gr (G.M.); kechagaa@chem.auth.gr (A.K.); 2 Department of Chemistry, University of Ioannina, P.O. Box 1186, 45110 Ioannina, Greece; 3 Biohellenika Biotechnology Company, Leoforos Georgikis Scholis 65, 555 35 Thessaloniki, Greece; annamichop@yahoo.gr

**Keywords:** 3D printing, chitosan, pectin, hydrogels

## Abstract

In the present study, chitosan (CS) and pectin (PEC) were utilized for the preparation of 3D printable inks through pneumatic extrusion for biomedical applications. CS is a polysaccharide with beneficial properties; however, its printing behavior is not satisfying, rendering the addition of a thickening agent necessary, i.e., PEC. The influence of PEC in the prepared inks was assessed through rheological measurements, altering the viscosity of the inks to be suitable for 3D printing. 3D printing conditions were optimized and the effect of different drying procedures, along with the presence or absence of a gelating agent on the CS-PEC printed scaffolds were assessed. The mean pore size along with the average filament diameter were measured through SEM micrographs. Interactions among the characteristic groups of the two polymers were evident through FTIR spectra. Swelling and hydrolysis measurements confirmed the influence of gelation and drying procedure on the subsequent behavior of the scaffolds. Ascribed to the beneficial pore size and swelling behavior, fibroblasts were able to survive upon exposure to the ungelated scaffolds.

## 1. Introduction

Chitosan (CS) is a linear seminatural polysaccharide derived from chitin through alkali deacetylation [1]. The abundance of chitin in nature alongside the simplicity of its deacetylation procedure renders CS a low-cost polymer [2]. CS consists of N-glucosamine and N-acetylglucosamine units, which are connected through β(1–4) glycosidic bonds [3]. It is a biocompatible, biodegradable and nontoxic polymer [4] with enhanced antimicrobial [3] and antioxidant activity [5]. Furthermore, due to its cationic nature, it has excellent mucoadhesive properties and in combination with its exceptional gel forming ability [2,3], it is a polymer extensively used in various applications including drug delivery and tissue engineering.

Depending on the desired application, CS has been prepared in different types of constructs, including porous scaffolds [6], membranes [7], beads [8] and nanoparticles [9]. When intended for biomedical applications and especially tissue engineering and wound healing, CS is usually processed with electrospinning [10], freeze drying [6], gas foaming, phase separation [6], solvent casting and electrophoretic deposition [11]. Each of these techniques has its limitations, and some apply to all of them; lack of control of the microstructure like shape and porosity, use of solvents and inability to produce complex structures are some of them [12]. With the evolution of 3D printing and the availability of cost-effective 3D printers on the market, the research on 3D printed constructs of natural polymers for biomedical applications including skin regeneration and engineering applications has bloomed [13,14]. 3D printing offers control over the size, shape and microstructure and allows the direct incorporation of cells to produce cell-laden scaffolds. Among the different 3D printing technologies, natural polymers, and especially CS, have been 3D printed with extrusion-based, fused-deposition modeling and solvent dispensing methods [12,15].

3D printing of CS still shows some limitations. There is still a need to improve its printability and fidelity by examining new combinations of materials that will result in optimized constructs [12]. Like for all bioinks, viscosity needs to be adjusted so that it can be easily extruded without clogging the output and maintain its shape afterwards. To improve printability, CS is often combined with other materials, like PEG [16], raffinose [17,18] and gelatin [19,20,21]. Cleymand et al. [22] developed CS/guar gum inks for 3D printing, resulting in enhanced dimensional stability of the printed patterns. Rahimnejad et al. [23] extensively studied CS-based thermosensitive hydrogels combined with different gelling agents and gelation kinetics, shear thinning and shear recovery behavior along with time and temperature dependence were taken into account, concluding in auspicious results. However, as most hydrogels, the stability of 3D printed CS constructs is often poor, and some type of crosslinking is applied either pre- or postprinting. These include photo-crosslinking, which requires the functionalization of CS [24], covalent crosslinking (e.g., genipin) [16], or physical crosslinking [17,18,19,25,26,27]. Adhikari et al. [28] developed a CS/alginate 3D printable bioink reinforced with hydroxyapatite, combining both pre- and post-printing crosslinking. Moreover, some studies reported the use of a support frame, made from synthetic thermoplastic polymer, to improve the shape of the CS constructs [29,30].

Derived from fruits and vegetables, pectin (PEC) is an anionic polysaccharide with good gelling properties [31]. It is biocompatible, biodegradable PEC has been used extensively in the food and pharmaceutics industries, and it can be 3D printed [32]. PEC possesses carboxyl groups that can physically crosslink with the amino groups of CS via the formation of polyelectrolyte complexes (PECs) in the pH range 3–6 [33]. Materials containing CS and PEC have been evaluated in vivo on rats [34] as well as in vitro on human stem cells, revealing encouraging results [35]. They were assessed as suitable candidates for tissue engineering applications, counting skin and bone regeneration, as they exhibited viscoelastic behavior, good mechanical properties and no cytotoxicity [33,36,37]

In this study, hydrogel scaffolds containing CS and PEC were prepared with pneumatic extrusion 3D printing, as a preliminary evaluation of their suitability for biomedical applications. The combination of CS and PEC was chosen to (i) improve the printability of both and (ii) stabilize the constructs via the formation of a PEC. Postprinting physical crosslinking with different bases was tested to further improve the stability of the constructs. The interactions between the components of the scaffolds were examined with FTIR spectroscopy, and their physicochemical properties were evaluated, including water swelling ratio, enzymatic hydrolysis rate and in vitro cytotoxicity.

## 2. Results and Discussion

### 2.1. Rheological Evaluation

**Figure 1:**
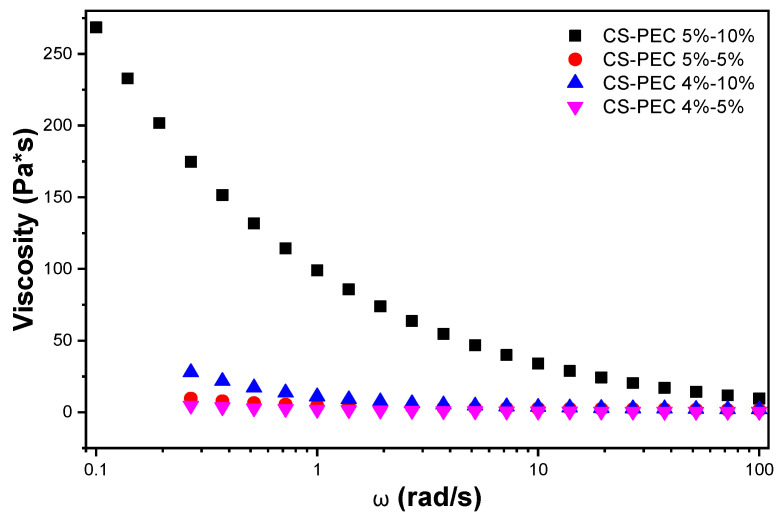
Viscosity dependency of the samples CS-PEC 4–5%, CS-PEC 4–10%, CS-PEC 5–5% and CS-PEC 5–10% at different shear rates.

The definition of rheology is the deformation of the matter’s flow and is investigating its response to an applied stress or strain [38]. The preparation of bioinks with specific printable behavior depends on the flow ability of the bioinks and, consequently, the assessment of their rheological behavior is essential. CS is a natural polymer whose rheological behavior depends mainly on the concentration of the examined solution [39]. Its viscosity increases while increasing the concentration of the ink. According to Kienzle-Sterzer et al. [40], CS gels have a shear thinning behavior. Consequently, an increase in shear stress would provoke a decrease in the viscosity value of the samples. PEC is a polysaccharide utilized extensively in food industry as a gelling agent. It has been utilized in bioinks preparation due to its ability to act as a thickening agent capable of increasing viscosity. According to Owens et al., while heating PEC solutions, in the temperature range between 0 °C to 50 °C, there is no detection of thickening of the solution. Contrarily, during cooling, hydrogen bonding is enhanced, providing strong gels [41]. The behavior of PEC is in accordance with our everyday experience. PEC is extensively utilized in jam preparation where this phenomenon takes place. The jam containing the polysaccharide is heated, PEC is solubilized and during cooling procedure, the viscosity of the jam increases since PEC is forming thick gels [42]. As a result, in a warmed PEC solution, viscosity along with storage modulus (G′) and loss modulus (G″) are expected to increase during cooling [43]. The shear thinning behavior of the prepared CS-PEC bioinks is clearly depicted in Figure 1 since the viscosity values decrease while increasing the rotation speed. According to the literature, shear thinning behavior is mandatory for a continuous flow during 3D printing procedure [22]. Interesting is the effect of PEC on the rheological behavior of the samples. A great difference in the viscosity values of CS-PEC 5–5% and CS-PEC 5–10% is noticed, confirming the thickening ability of PEC.

Hydrogels are considered suitable for 3D printing only if they are capable of forming cylindrical fibers during extrusion and retaining distinct layers throughout the printing procedure [44]. Inks with viscosity values exceeding 10,000 Pa⋅s are characterized as too viscous, forming usually nonuniform filaments with difficulty in depositing smoothly; however, they are characterized by excellent printing accuracy. On the contrary, inks with viscosity values lower than 100 Pa⋅s are characterized as too fluidic, showing great extrudability but poor shape fidelity [45,46]. However, the utmost importance of the printed hydrogels is their ability to retain their shape during the printing procedure. According to the literature, an optimal range of viscosity suitable for high fidelity printing was found to be between 400–4000 Pa⋅s [47].

**Figure 2:**
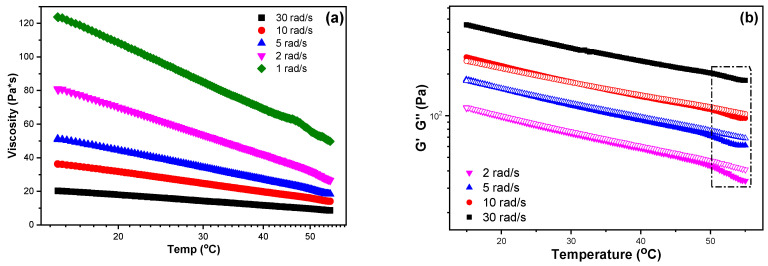
(**a**) Viscosity and (**b**) storage modulus (G′, infilled symbols) and loss modulus (G″, hollow symbols) dependency over temperature at fixed frequencies of the sample CS-PEC 5–10%.

Among the four prepared inks, the sample CS-PEC 5–10% revealed the higher, comparable to bibliography viscosity values and, consequently, its rheological behavior was further assessed. Figure 2a clarifies the change of the sample’s viscosity between 15–55 °C for different shear stress values. During cooling, the viscosity is increased in every applied rotational speed, behavior which is expected according to the individual polymers’ rheological performance. Moreover, viscosity values are higher for lower frequencies. In Figure 2b, the storage (G′) and loss modulus values (G″) of the sample CS-PEC 5–10% between 15–55 °C for various fixed frequencies are presented. A sol-gel transition at 53 °C is detected during cooling, while the inks behave like weak gels in the temperature range 15–50 °C, confirming once again the improved mechanical properties of the inks.

### 2.2. Morphological Characterization

**Figure 3:**
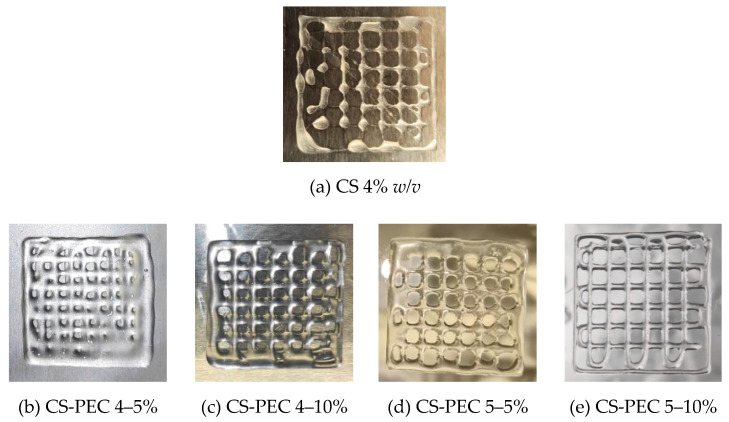
Photos of 3D printed (**a**) CS solution 4% *w*/*v* and of the samples (**b**) CS-PEC 4–5%, (**c**) CS-PEC 4–10%, (**d**) CS-PEC 5–5% and (**e**) CS-PEC 5–10%.

According to the literature, 3D printing of natural polymers is still challenging since these water-soluble polymers are not able to maintain a concrete structure [48]. A square grid was selected for 3D printing since according to Ma et al. [49], this scaffold facilitates fibroblasts proliferation and skin regeneration, as compared to aligned and randomly distributed scaffolds. Figure 3a presents a 3D printed scaffold of neat CS 4% *w*/*v* solution. CS viscous solutions (25–30 wt %) extruded under high pressure results in high-fidelity printed scaffolds [50]. However, in lower concentrations, the ink’s inability to form uniformly extruded filament, results in a dot-structure instead of a uniform grid. CS inks with high viscosity values (~1500 Pa⋅s) result in high fidelity microstructures [48]. Figure 3b–e present the 3D printed scaffolds of the CS-PEC inks. As mentioned above, the viscosity values of the inks CS-PEC 4–5%, CS-PEC 4–10% and CS-PEC 5–5% are low and the resulted shape fidelity of the final scaffolds is insufficient. However, the effect of PEC on the printing ability of the inks, as well as on the final scaffolds’ shape, is obvious. While increasing PEC concentration, the ability of inks CS-PEC 4–10% and CS-PEC 5–10% to maintain their morphology is evident, resulting in regular grids with square holes. Moreover, the higher CS concentration results in increased viscosity ink, which, in conjunction with the presence of PEC, renders the CS-PEC 5–10% the most appropriate among the four CS-PEC inks.

**Figure 4:**
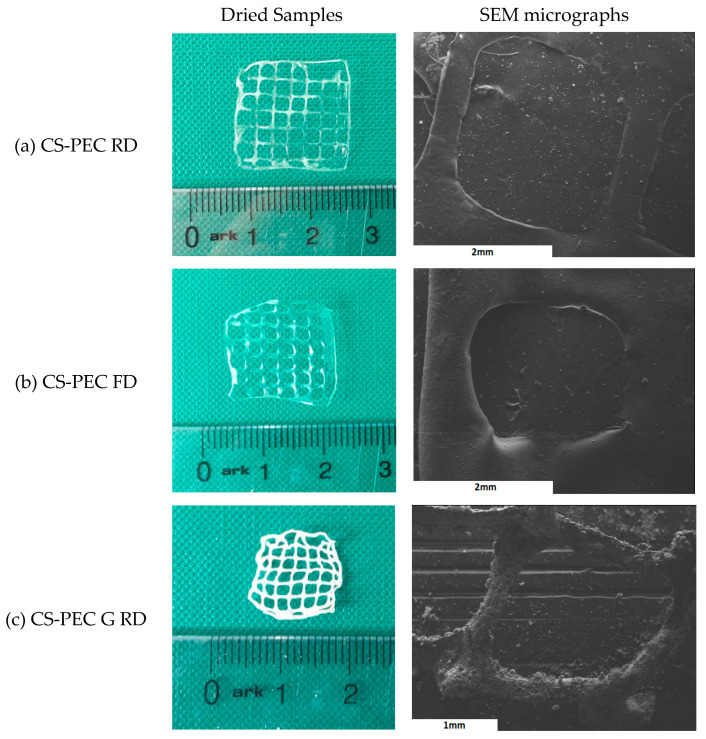

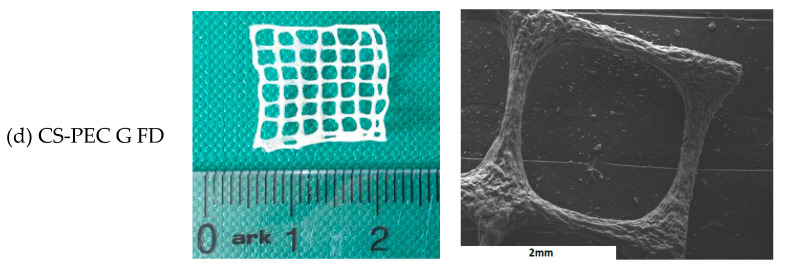
Photos of dried samples and SEM micrographs of the printed scaffolds (**a**) CS-PEC RD, (**b**) CS-PEC FD, (**c**) CS-PEC G RD and (**d**) CS-PEC G FD.

Macroscopic photographs and scanning electron microscope micrographs of the dried samples are shown in Figure 4. The different postprinting gelating and drying conditions applied on the samples resulted in four different samples CS-PEC RD, CS-PEC FD, CS-PEC G RD and CS-PEC G FD (G; gelation, RD; drying in room temperature, FD; freeze drying). Drying of the scaffolds is important since their storage and transfer are easier in comparison to wet scaffolds. As it can be observed, the samples CS-PEC RD and CS-PEC G FD retain their shape and morphology macroscopically whereas shrinkage is observed in the samples CS-PEC FD and CS-PEC G RD during drying and storage. The evaluation of drying procedure is important since according to Claymand et al. [22], drying after printing procedure might induce shrinkage of the scaffolds, whereas their inner pore structure affects the proliferation of cells on them [37]. As expected, ungelated samples are transparent, which is in agreement with literature data [51]. In contrast, gelation of the scaffolds with alkali solution (10 min gelation time) results in opaque brittle samples where according to Frick et al. [52], the alkali treatment of CS films is responsible for their increased brittleness. Furthermore, it is commonly known that subjecting natural polymer solutions in lyophilization results in porous structures [53]. Consequently, through these images the formed porous structure of the scaffolds CS-PEC FD is revealed, in contrast with the samples RD, attributed to the sublimation of the contained water. This result is in agreement with previous data from our group, where ultraporous structures of CS dressings were prepared with lyophilization [54]. The CS-PEC G FD sample shrunk less than CS-PEC G RD, likely due to the stabilization of its structure during its storage in the freezer before its lyophilization. Furthermore, SEM micrographs depict the microstructure and the morphology of the printed scaffolds. Concerning the ungelated samples, their surface is smooth, whereas the alkali-treated scaffolds’ surface is rougher, ascribed to the gelation with KOH solution.

**Figure 5:**
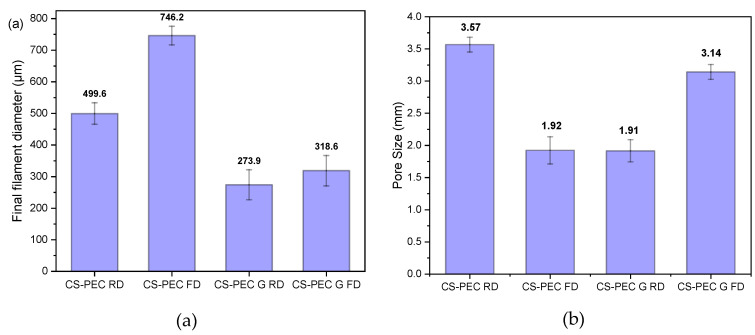
Average (**a**) filament diameter and (**b**) pore size of the samples CS-PEC RD, CS-PEC FD, CS-PEC G RD and CS-PEC G FD. 5 measurement were performed for each sample.

Through SEM micrographs, the average filament diameter and the average pore size were measured (Figure 5a,b). Concerning the diameter of the filament, it is observed that gelated samples CS-PEC G RD and CS-PEC G FD have smaller average diameter, 273.9 μm and 318.6 μm, respectively, while ungelated scaffolds present larger diameter. The sizes of the gelated scaffolds’ diameters are comparable to the needle’s inner diameter (260 μm). This behavior is expected and attributed to the maintenance of the 3D structure after the instant postprinting gelation of the samples. However, the ungelated scaffolds have a liquid-like behavior and they present a tendency where upper and lower layers fuse together, resulting in the distortion of the scaffolds’ shape. Regarding the obtained pore size, the scaffolds with larger diameter naturally present a smaller pore size and vice versa. The CS-PEC G RD sample deviates from the aforementioned behavior by having small pore size with small filament diameter. However, the scaffold’s shape shrunk after the gelation and drying procedures. The sample CS-PEC G FD was the only one the retained its shape having large pore size and small filament diameter after printing, crosslinking and drying.

### 2.3. Characterization of the Scaffolds

**Figure 6:**
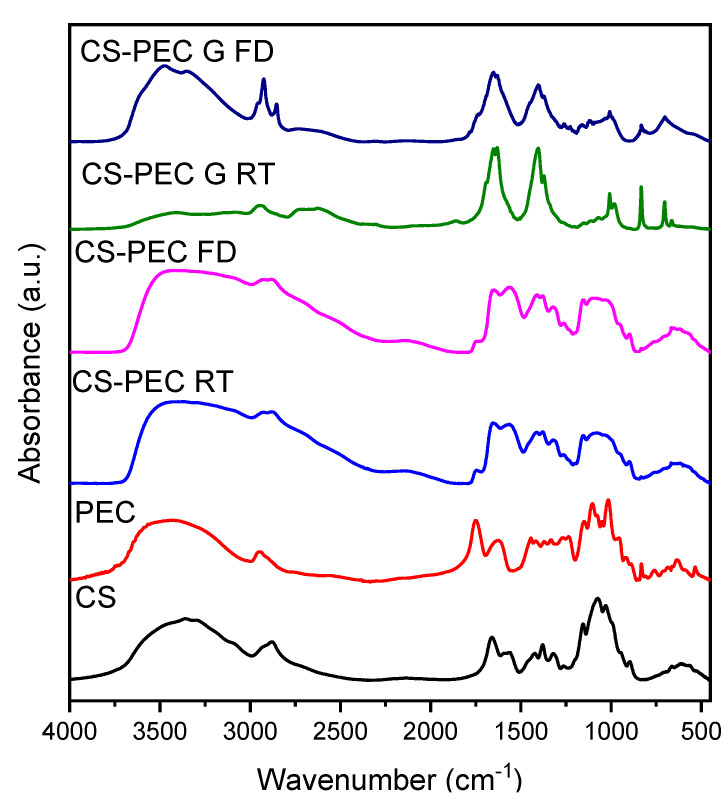
FTIR spectra of the 3D printed samples CS-PEC RD, CS-PEC FD CS-PEC G RD and CS-PEC G FD.

The addition of PEC to the CS solution aims for the preparation of bioinks with improved printability. However, the presence of both polysaccharides, results in intermolecular interactions. The characteristic FTIR bands of CS and PEC as well as of the formulated scaffolds are presented in Figure 6. Briefly, the typical bands of CS are present at 3400 cm^−1^ due to the O-H hydroxyl groups, at 3360 cm^−1^ attributed to -NH group stretching vibrations whereas at 1656 cm^−1^ and 1584 cm^−1^ are the peaks corresponding to amide I and II, respectively [55]. The main absorbance bands characterizing PEC are present at 3436 cm^−1^ attributed to O-H groups, at 2945 cm^−1^ ascribed to symmetric -CH_3_ stretching, while the bands at 1747 and 1630 cm^−1^ are owed to the stretching of the carbonyl groups C=O of the carboxylic and ester moieties, respectively [56]. The FTIR spectra of the CS-PEC scaffolds reveal the presence of both polysaccharides, while small shifts in the characteristic bands of the polymers are detected. These shifts are attributed to interactions owed to the presence of hydrogen bonds and to electrostatic interactions between the anionic carboxylic groups of PEC and the positive charged amino groups of CS. According to the literature, during the formation of polyelectrolyte complexes, the main changes are detected in the range of 1800–1600 cm^–1^, providing evidence of the interaction of the amino and carboxyl groups. Due to the formation of intermolecular ionic bonds, the asymmetric stretching vibration of the carbonyl group of the carboxylate (COO–) groups in pectin along with the bands ascribed to asymmetric and symmetric bending vibrations of the NH_3_ groups are expected to be shifted [57,58]. More specifically, in the CS-PEC RD and FD samples, the characteristic broad peaks attributed to hydroxyl and amino groups are shifted to 3462 cm^−1^, whereas the peaks attributed to amide I, II as well as to the carbonyl groups are all shifted to lower wavenumbers. The spectra of CS-PEC G RD and CS-PEC G FD are interesting; the vibration of the carbonylic bond at 1747 cm^−1^ is shifted to 1738 and 1698 cm^−1^ at the CS-PEC G FD and G RD spectra, respectively, whereas the peaks of the amides I and II are not clearly distinguished, due to the gelation of the samples with KOH solution.

**Figure 7:**
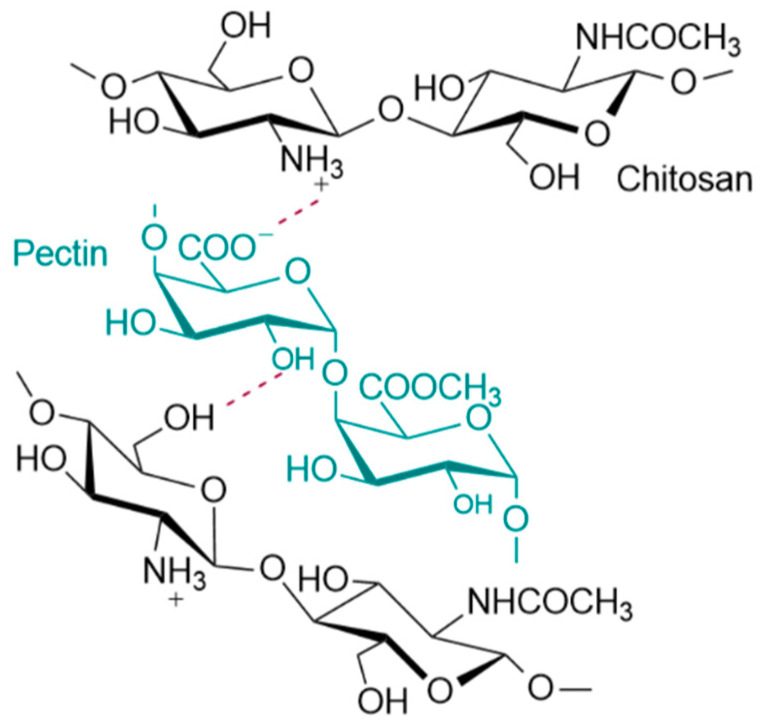
Possible interactions between the end groups of CS and PEC.

The formation of polyelectrolyte complexes between CS and PEC occurs in the pKa range of the two polymers. The pKa value of CS lies between 6.2–7.0 whereas PEC’s pKa value is between 3.5–4.5. The pH value of the prepared CS-PEC inks was measured to be in the range of 4.0–4.5. Consequently, the amino groups of CS and the carboxylic groups of PEC are positively and negatively charged respectively, leading to the formation of H-bond interactions among the polysaccharide networks as depicted in Figure 7. When the degree of esterification of PEC is high (>50%), the interactions occurring between carboxyl and amino groups in semidilute and gelling solutions, are intermolecular associations governed by hydrogen bonds and hydrophobic interactions [58,59]. These intermolecular interactions are leading to the enhanced printing behavior of the ink in comparison to the printing behavior of neat CS solution as well as to the ability of the 3D printed scaffolds to maintain their shape after the printing procedure.

**Figure 8:**
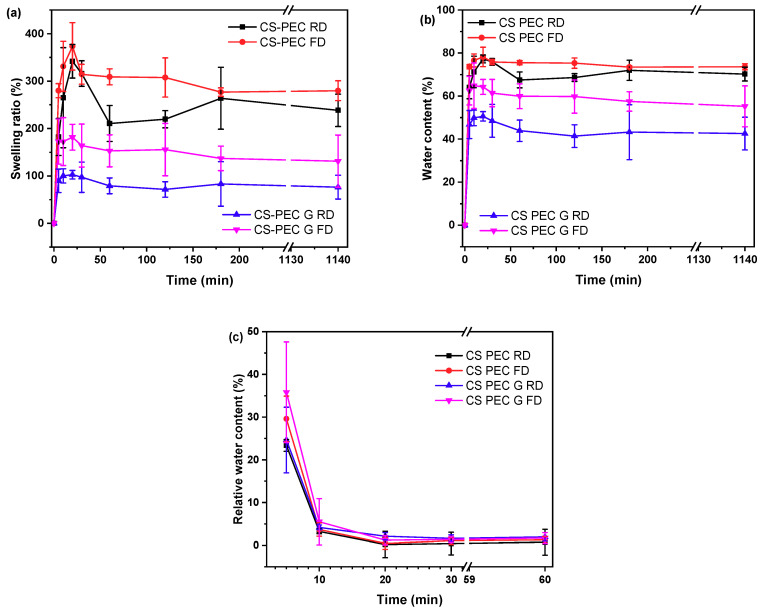
(**a**) Degree of swelling, (**b**) water content and (**c**) dehydration of the samples CS-PEC RD, CS-PEC FD, CS-PEC G RD and CS-PEC G FD as a function of time.

Polymeric scaffolds addressed for tissue engineering applications must meet various requirements, namely biocompatibility, good mechanical properties and appropriate pore size [60]. Among them, an important characteristic is their ability to swell in aqueous media. When scaffolds are in contact with human tissues, increased amounts of body fluids are absorbed [61]. CS and PEC, as natural polysaccharides, have an innate ability of swelling in aqueous solutions. The gelation of the scaffolds, as well as the utilized drying procedure affects their swelling ability. The swelling behavior of polymeric scaffolds is a pH-dependent phenomenon that reaches an equilibrium point within the first 2 h [62]. Padney et al. concluded that when the ionic interactions between CS and PEC are weaker, the swelling ability of the polymeric matrixes is higher [63]. More specifically, in pH 7.4, the amine groups of CS are partially deionized, whereas the carboxylic groups of PEC are negatively charged. Consequently, an enhanced swelling ability of the printed scaffolds in pH 7.4 is expected. Figure 8a presents the swelling behavior of the 3D printed scaffolds in SBF buffer while Figure 8b shows the water content of the scaffolds. As depicted, all the scaffolds have an initial burst water uptake during the first 20 min, followed by a small reduction of the amount of the swelled water, possibly due to erosion of the scaffolds, reaching an equilibrium point at 3 h. Ungelated scaffolds CS-PEC RD and CS-PEC FD present higher swelling ability up to 341 ± 35% and 373 ± 50%, respectively, in contrast to gelated CS-PEC G RD and CS-PEC G FD samples, which swell up to 102 ± 9% and 181 ± 27%, respectively. The water content of the scaffolds is CS-PEC RD 77 ± 1.8%, CS-PEC FD 78 ± 4.5%, CS-PEC G RD 50 ± 2.2% and CS-PEC G FD 64 ± 3.5%. The samples’ behavior is in agreement with literature data, since treatment with alkali in CS scaffolds leads to reduced swelling ability [52,64]. Furthermore, freeze dried samples tend to have higher swelling ability and water content in comparison to room temperature dried scaffolds, revealing the effect of the increased porosity caused by the freeze-drying procedure on the swelling properties. Naturally, neat CS presents a low degree of swelling which varies between 50–150% depending on the pH, the molecular weight and the degree of deacetylation [65]. Consequently, the scaffolds’ enhanced swelling is ascribed to the presence of PEC, since the anionic end groups enhance the electrostatic repulsions, ameliorating the total swelling ability of the scaffolds.

An important characteristic of polymeric scaffolds for skin regeneration applications is their dehydration ability which is also referred as swelling reversibility. Swelling reversibility is interlaced to the reusability of the scaffolds [66]. Figure 8c shows the relative water content of the samples and their behavior through the dehydration process. The freeze-dried samples CS-PEC FD and CS-PEC G FD had higher relative water content in *t* = 0 min (36 ± 5.3% and 30 ± 11.8%, respectively) owed to their higher porosity, whereas CS-PEC RD and G RD relative water content was 23 ± 1.4% and 24 ± 7.7%, respectively. During the first 5 min, the water content of all samples diminished to 3–4%, while at 60 min, the water content is 0.7–2%. Developing reusable scaffolds with swelling ability and thereafter almost complete dehydration, is, according to the literature, helpful for long-term applications on wounds [67].

**Figure 9:**
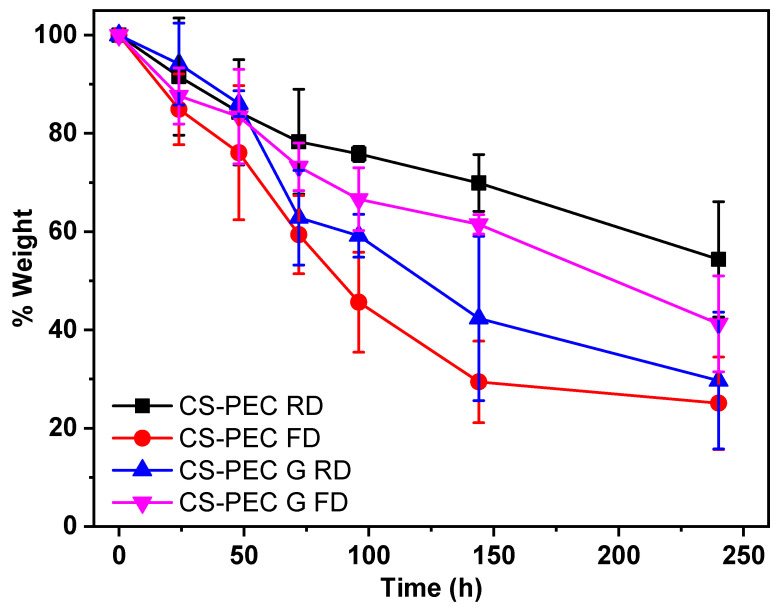
Mass loss during enzymatic hydrolysis of the samples CS-PEC RD, CS-PEC FD, CS-PEC G RD and CS-PEC-G FD.

CS is able to be depolymerized by lysozyme through the hydrolysis of its glycosidic bonds [68]. According to the literature, the weight loss of CS scaffolds depends on the polymers’ concentration of the initial solutions, the degree of deacetylation (DD) along with the molecular weight and their swelling behavior [69]. Since molecular weight, concentration and DD are constant in CS-PEC scaffolds, their hydrolysis behavior changes in accordance with their swelling ability. As the aqueous enzymatic solution reaches the polymeric scaffold, lysozyme begins to break down the polymer. Consequently, the higher porosity allows more surface area for the enzyme degradation to take place. Figure 9 presents the mass loss results of the samples during enzymatic hydrolysis. Ungelated samples behave as expected and described in the literature, since increased swelling ratio results in increased degradation rate [37]. The sample CS-PEC FD, presenting the higher swelling ability, lost approximately 80% of its mass during enzymic hydrolysis, while CS-PEC RD lost 45% of its mass. Interesting is the hydrolysis behavior of the gelated samples. The gelation of the scaffolds induced reduction of the mesh size of the polymeric network, likely leading to prevention of lysozyme effectively cleaving the glycosidic bonds. As a result, the mass loss of the samples CS-PEC G FD and CS-PEC G RD is up to 60% and 70%, respectively.

**Figure 10:**
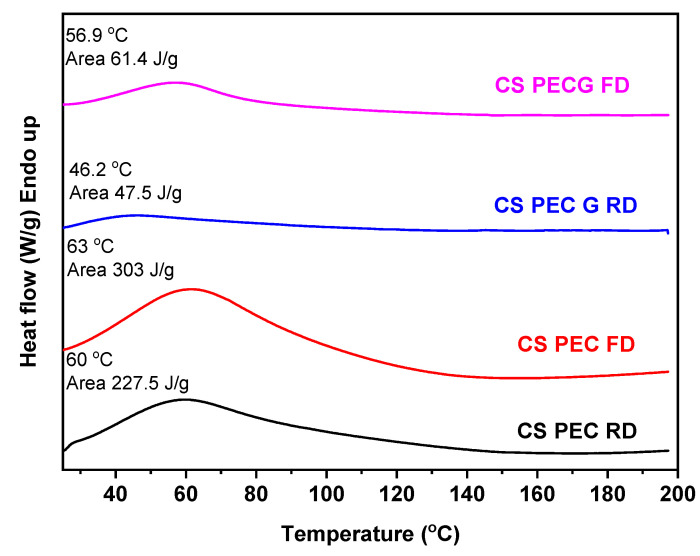
DSC curves of CS-PEC RD, CS-PEC FD, CS-PEC G RD and CS-PEC G FD.

The thermal behavior of the prepared scaffolds was evaluated through DSC measurements. CS, according to the literature, has a characteristic endothermic peak at around 50–100 °C. This temperature range in the CS thermogram is also called dehydration temperature and is attributed to the loss of the absorbed water due to the presence of hydrophilic groups on the CS backbone [70]. Figure 10 presents the DSC scans of the CS-PEC scaffolds. It is evident that all the 3D printed scaffolds have an endothermic peak around 60 °C accredited to their dehydration. Interesting are the enthalpy values of these endothermic peaks, since higher values mean greater amount of absorbed water in the scaffold. The ungelated samples CS-PEC RD and CS-PEC FD have grater enthalpy in comparison to gelated CS-PEC G RD and CS-PEC G FD. Concurrently, freeze-dried samples CS-PEC FD and CS-PEC G FD reveal higher enthalpy than the room dried CS-PEC RD and CS-PEC G RD. These results are in agreement with the swelling and dehydration measurements, where gelation and inner porosity affect the absorbed moisture of the scaffolds.

**Figure 11:**
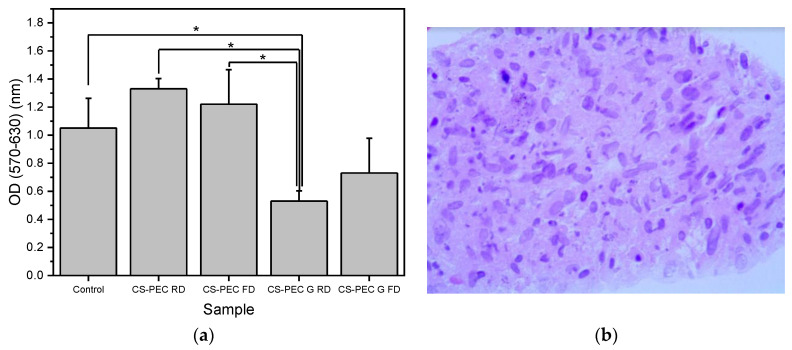
(**a**) MTT assays results on the proliferation of fibroblasts exposed to the scaffolds. Mean ± SD. * *p* ≤ 0.05, (**b**) Hematoxylin-and-eosin-stained image of fibroblasts seeded on the CS/Pec RD construct.

Polymeric scaffolds for skin regeneration applications must allow the attachment and proliferation of cells. Fibroblasts are cells mainly responsible for collagen production, which is the major components of the extracellular matrix of the dermis [71]. According to Howling et al., CS has a positive impact on fibroblast proliferation, as well as on the contraction of collagen lattices [72]. Furthermore, the morphology of a scaffold has been proven to drastically affect the growth of various cell types [73]. The porosity along with the 3D structure of a scaffold has a great impact on cell attachment, affecting cellular growth [74,75]. In the present study, the viability and cytocompatibility of fibroblast cells on the CS-PEC scaffolds was assessed, aiming to ensure their viability upon exposure to the materials through an MTT assay (Figure 11a). According to the literature, CS-PEC matrices reveal no cytotoxicity [37,58]. Ungelated samples CS-PEC RD and CS-PEC FD behaved accordingly and displayed no cytotoxicity, while the absorbance is not significantly different from the control sample. In contrast, gelated samples CS-PEC G FD and CS-PEC G RD revealed lower values when compared with the control sample. This result is in contrast with the data reported by Bergonzi et al. [18], where CS-PEC scaffolds gelated with KOH solution were able to support cell growth. According to Kruse et al., fibroblasts could adhere and proliferate in slightly alkaline conditions [76]. However, in basic pH environment, they show increased apoptosis [77]. Consequently, as the gelation was conducted with KOH solution, the pH value of the scaffolds was too basic for the cells and a reduction of the living population was expected. A further confirmation of the suitability of the polymeric scaffolds supporting the adherence and proliferation of fibroblasts was evaluated. Figure 11b presents cells distribution within the scaffolds in histological sections. It is evident that cells could adhere and proliferate all over the scaffold CS-PEC RD. Even though the data presented are preliminary, however, the successful seed of fibroblasts on the scaffold along with the cell distribution within the scaffold in histological sections is evidence that the scaffold CS-PEC RD could be further assayed for application in tissue engineering and eventually for other biomedical applications.

## 3. Materials and Methods

### 3.1. Materials

Chitosan with high molecular weight (310,000–375,000 Da) and a degree of a deacetylation >75% was supplied from Sigma Aldrich Co (St. Louis, MO, USA). Pectin from citrus peel with molecular weight 30,000–100,000 and a degree of esterification 63–66% (high methoxyl Pectin) was obtained from Fluka Chemie GmbH, Buchs, Switzerland. Potassium hydroxide was purchased from Merk (KGaAn Darmstadt, Germany). Lysozyme from chicken egg white was supplied from Sigma Aldrich Co (St. Louis, MO, USA). All the other reagents utilized were of analytical grade.

### 3.2. Preparation of the CS-Pectin Solutions and Scaffolds

For the preparation of CS-PEC solutions, CS and PEC powders were mechanically mixed and suspended in a proper amount of water under mechanical stirring. Acetic acid was added, resulting in the formation of a 2% *v/v* acetic acid aqueous solution. In the acidic pH, CS was solubilized, while thereafter, temperature was increased to 80 °C and PEC was completely dissolved. During cooling, the solutions turned into gels at 50 °C. The final mass ratio of the samples was CS:PEC 10:1 and 20:1, while the total concentration was 4.2% *w*/*v*, 4.4% *w*/*v*, 5.25% *w*/*v* and 5.5% *w*/*v* for the samples CS-PEC 4–5%, CS-PEC 4–10%, CS-PEC 5–5% and CS-PEC 5–10%, respectively (Table 1). These are the lowest polymeric concentrations where the ink was easily extruded from the nozzle with a continuous flow and the printed scaffolds retained their shape. Afterward, the samples were placed under vacuum to remove the air bubbles generated while stirring. When a homogenous gel was formed, it was poured into a jet dispenser’s nozzle syringe suitable for 3D printing.

**Figure 12:**
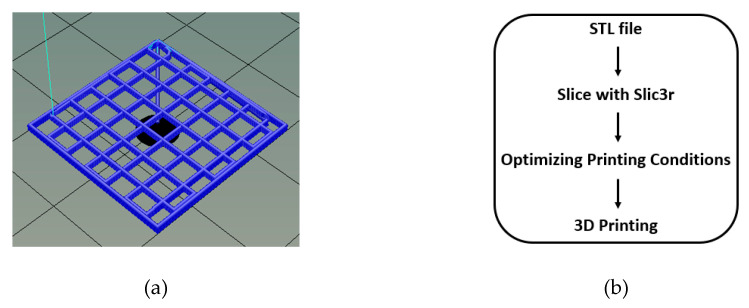
(**a**) 3D model of the constructs with dimensions 2 × 2 × 0.1 mm and after slicing with Slic3r (6 layers) and (**b**) process flow of file preparation for 3D printing.

The CS-PEC solutions were extruded pneumatically by an extrusion-based 3D Bioprinter (CELLINK^®^ Inkredible, Gothenburg, Sweden), through a nozzle of inner diameter 0.26 mm (G25). An STL file of a three-dimensional rectangle was utilized for the 3D printing while the slicing of the STL sample was performed with Slic3r software (infill 80%, 6 layers, angle of layers 0°) (Figure 12a,b) [78,79]. Printing conditions were optimized according to the concentration of the ink, and they are summarized in Table 1. The printed scaffolds obtained from the optimum ink composition (CS-PEC 5–10%) were dried and physically gelated. Drying of the samples was performed using two methods, by evaporation of the solvent in room temperature or by lyophilization. The gelation of the samples was performed post-printing by the addition of KOH 1.5 M solution on the constructs according to Bergonzi et al. [18]. Each sample was printed in multiple times for the various physicochemical measurements. Gelation time was measured optically, when the scaffolds became opaque and remained innate when inverting the petri dish [80]. The different drying and gelating conditions resulted in the production of four different samples, namely CS-PEC RD, CS-PEC FD, CS-PEC G RD and CS-PEC G FD, where G stands for gelation, RD stands for drying in room temperature and FD stands for freeze drying.

### 3.3. Rheological Evaluation of the Inks

Rheological evaluation was conducted according to Nordby et al. on a ARES (TA Instruments, New Castle, DE, USA) with a cone-plate geometry [81]. The cone angle was set at 1° while its diameter was 75 mm. Due to the morphology of the cone, there was a gap of 0.05 mm between the two flat surfaces. Each sample was subjected into stirring at a temperature above the gelling point of the samples, at 55 °C before the measurement. Silicone oil was applied to the free surface of the samples in order to avoid evaporation of the contained water. Equation (1) was utilized for the evaluation of the viscosity values.

(1) $$\left|\eta^{*}\right|=\left[{\left(\begin{array}{c} G^{\prime \prime }\\ \omega \end{array}\right)}^{2}+{\left(\begin{array}{c} G^{\prime }\\ \omega \end{array}\right)}^{2}\right]$$

### 3.4. Characterization of the Printed Scaffolds

#### 3.4.1. Fourier-Transformed Infrared Spectroscopy (FTIR)

The FTIR spectra of the samples were obtained by FTIR spectrometer (model FTIR-2000, Perkin Elmer, Waltham, MA, USA). In brief, a small amount of each sample was triturated with a proper amount of potassium bromide (KBr) and the disks were formed under pressure. The spectra were collected in the range from 400 to 4000 cm^−1^ at a resolution of 4 cm^−1^ using 16 coadded scans while the baseline was corrected and converted into absorbance mode.

#### 3.4.2. Scanning Electron Microscopy

Scanning electron microscopy (SEM) images were obtained with an electron microscope JEOL 2011 (Akishima, Tokyo, Japan). Each sample was placed on the holder and covered with carbon to provide good conductivity of the electron beam. Operating conditions were set at accelerating voltage 20 kV, probe current 45 nA and counting time 60 s.

#### 3.4.3. Swelling Capacity and Dehydration

Swelling ability of the prepared scaffolds was evaluated by measuring the amount of water sorption aptitude of simulated body fluid (SBF) buffer (pH = 7.2), prepared as described by Kokubo et al. [82]. The swelling ability was evaluated in the samples CS-PEC RD, CS-PEC FD, CS-PEC G RD and CS-PEC G FD. Each dry scaffold was carefully weighed (Wd) and washed twice with SBF for 10 min. The samples were then placed on filter paper in order to remove the excess surface water and their weight (W_f_) was measured at predetermined times (5 min, 10 min, 20 min, 30 min, 1 h, 2 h, 3 h and 48 h). Swelling ratio and water content were calculated according to equations (2) and (3) respectively.

Swelling ratio % = (W_f_ − W_d_) × 100/W_d_ (2)

Water content % = (W_f_ − W_d_) × 100/W_f_ % (3)

The dehydration progress of the samples was evaluated by measuring the water content loss of the samples. The samples were placed in water for 24 h (W_0_, water content 100%) and then placed in vacuum oven (40 °C, 200 mbar). The weight of the samples (W_f_) was measured in predetermined times (5 min, 10 min, 20 min, 30 min, 60 min). The measured weight was compared to the initial weight of the dry samples (W_d_, water content 0%). The relative water content was assessed through the equation (4). Measurements were performed in triplicate.

Relative water content = (W_f_ − W_d_) × 100/W_0_ − W_d_ (4)

#### 3.4.4. Enzymatic Hydrolysis

Enzymatic hydrolysis of the samples was evaluated by placing the samples in 5 mL of SBF, pH = 7.4 containing 1 mL of lysozyme solution (0.8 mg/mL). The samples were then placed in an oven at 37 °C and at predetermined times (0 h, 24 h, 48 h, 72 h, 96 h, 144 h and 240 h), they were washed with distilled water, vacuum dried in an oven at 50 °C and weighed. Measurements were performed in triplicate.

#### 3.4.5. Differential Scanning Calorimetry (DSC)

Thermal analysis studies were carried out by a Perkin-Elmer Pyris 6 differential scanning calorimeter (DSC) (Waltham, MA, USA) calibrated with indium and zinc standards in order to examine the crystalline state of the samples. About 5 mg of each sample were placed in sealed aluminum pans and heated up from 30 to 200 °C with a heating rate 20 °C/min in inert atmosphere (N_2_, flow rate 50 mL/min).

#### 3.4.6. In Vitro Cell viability

Scaffolds of cylindrical shape were printed with density of first layer 100%. To evaluate the direct cytotoxicity of our materials, the different CS-PEC scaffolds were deposited on confluent cell cultures. Normal human fibroblast cells isolated and expanded from skin biopsies as described elsewhere at passage 1 [83] were seeded at 22,500/well onto a 24-well plate. The cells were left to grow in DMEM (BIOWEST, Nuaillé, France), 10% fetal bovine serum (BIOWEST), 1% penicillin/streptomycin (BIOWEST) for 48 h and their viability was estimated by [3-(4,5-dimethylthiazol-2-yl)-2,5-diphenyl tetrazolium bromide] (MTT) assay at time 0 before treatment. Then, the four different scaffolds were added into each well containing cells in triplicates for 24 h and subjected to MTT assay. The viability of cells was evaluated by estimating the relative change in optical densities (indicative of the cell number) after 24 h of exposure and comparing this value among the scaffolds and against the plastic control. The experiment was performed in triplicate and the results were expressed as mean ± standard deviation (SD). Unless otherwise stated, one-way ANOVA with post hoc Tukey test was used. A *p*-value ≤ 0.05 was considered statistically significant.

The scaffold presenting the optimal results in this experiment was assessed for its capacity to support fibroblast adherence and expansion. Before seeding, the scaffolds were sterilized by immersion in 70% ethanol for 30 min. Then, the scaffold was calibrated by being incubated overnight at DMEM complete medium at 37 °C, 5% CO_2_. Fibroblasts were seeded at a density of 750,000 cells/cm^2^ onto a culture area of 0.47 cm^2^. Fibroblasts in the scaffold were allowed to grow for 3 weeks post-seeding by changing the culture medium, DMEM complete every other day and then fixed using an overnight incubation in 4% paraformaldehyde at 4 °C. Inserts were paraffin-embedded and sectioned followed by processing for hematoxylin and eosin (H&E) staining. Tissue sections were photographed at ×40 magnification to examine the distribution and growth of fibroblasts within the scaffold.

## 4. Conclusions

In the present study, two natural polysaccharides, CS and PEC, were utilized for the preparation of inks appropriate for 3D printing. Rheological analysis measurements confirmed the effect of PEC on the rheological behavior of the inks and established the applicability in the printing procedure of the sample CS-PEC 5–10% while optimum printing conditions were found. The effect of gelation and different drying conditions on the behavior of the 3D printed scaffolds were assessed. Through SEM micrographs the average pore size and filament diameter were measured whereas, FTIR spectra confirm the presence of intermolecular interactions between the two polymers. Swelling and hydrolysis studies verified the effect of gelation and freeze-drying procedure on the subsequent behavior of the scaffolds. Finally, the viability of fibroblasts on the CS-PEC scaffolds was estimated and ungelated scaffolds are proved to successfully support their proliferation.

## Figures and Tables

**Table 1:** Optimum printing parameters of the inks.

Sample	Final Polymeric Concentration	CS:Pec	Infill	Speed (m/s)	Pressure (kPa)	Temperature
CS-PEC 4–5%	4.2% *w*/*v*	20:1	80%	2.5	110	RT
CS-PEC 4–10%	4.4% *w*/*v*	10:1	80%	2.5	260	RT
CS-PEC 5–5%	5.25% *w*/*v*	20:1	80%	3	180	RT
CS-PEC 5–10%	5.5% *w*/*v*	10:1	80%	3	285	RT

## Data Availability

Data is contained within the article.
